# Mapping the Way to Good Health: The Interdisciplinary Challenges of Geographers in Medical Research

**DOI:** 10.3390/ijerph191912419

**Published:** 2022-09-29

**Authors:** Richard Casey Sadler, Kristian Larsen

**Affiliations:** 1 Division of Public Health, College of Human Medicine, Michigan State University, Flint, MI 48502, USA; 2 Department of Family Medicine, College of Human Medicine, Michigan State University, East Lansing, MI 48824, USA; 3 CAREX Canada, School of Population and Public Health, University of British Columbia, Vancouver, BC V6T 1Z4, Canada; 4 Department of Geography and Planning, University of Toronto, Toronto, ON M5G 1G6, Canada; 5 Department of Geography and Environmental Studies, Toronto Metropolitan University, Toronto, ON M5B 2K3, Canada

**Keywords:** medical geography, geographic information systems, interdisciplinary research, place effects on health, social determinants of health, public health

## Abstract

Geography has an important role to play in shaping the direction of medical research. In particular, its tools and theory provide essential understanding to the impacts of place on health behaviors and outcomes. Understanding some of its evolution—particularly into the subfield of medical geography—is therefore useful both for geographers and medical researchers. In this paper, we present some of the debates that geographers have grappled with, the growth of GIS (particularly in the context of medical research), some important methodological considerations that geographers help center, and some recommendations for future work at this nexus. Throughout, we speak from the perspective of geographers who have worked nearly exclusively in the health sciences since obtaining our PhDs.

## 1. Introduction

Geography’s existence as a discipline has long been debated in academic circles. Much of this stems from questions of whether geography is merely a collection of methods, the extent to which geographical theories offer a coherent explanation for its existence, and how interdisciplinary work is premised within the discipline or on collaborating with geographers. Consider how multi-disciplinary spatial scientists are trained, and what they experience in the world of public health and environment research. Indeed, even the title of this journal—and the handling of this Special Issue by two individuals trained in geography but working in medical research—suggests a potential gap between training and practice. While it is not our intention here to replicate that debate, we lay out a brief perspective on geography’s evolution, and specifically how medical geography (and the tools of geographic information science, or GISci) grew both independently of and alongside the medical field. This is an importance distinction, of course, because medical geography itself it not ‘medical’ in the sense that it normally deals with the diagnosis or treatment of disease (a reason others have used to describe it as ‘epidemiologic geography’ or ‘the geography of disease’) [1]. We also explore how the methods and approaches of geography give voice and nuance to concepts within public health that may otherwise be inadequately described. Both authors were geographers whose work took them into the medical or population health field. Our situations provide a keen perspective on the interdisciplinary linkages found at the nexus of geography, GISci, medicine, and population health.

## 2. The Evolution of Geography

Given geography’s long grappling with its own existence, it bears recounting some of this evolution. Common themes have been present in the characterization of geography through time. Lukerman [2] offered a description of geography as a discipline whose questions are inherently geographic—that it investigates the very thing it is about. Harvey ties in the close connection of humanism to the discipline, saying that “in order to change the world, we have first to understand it” [3] (p. 23). Robinson followed this thought, remarking how “geographers have attempted to know as much about an area or a region as possible” [4] (p. 524). Baerwald gave a thoughtful exploration of the common traditions of geography through time, recounting Pattison, Taaffe, Abler, Salter, and Natoli’s overlapping conceptualizations of geography as inherently about place [5]. Throughout, many have remarked on the centrality of the study of space and place, and/or on human-environment interactions in the discipline of geography [2,4,5,6].

Some critical theorists in the later part of the 20th century expressed various concerns about geography, including on its ‘soft centre’ [7], and on the flaws of a colonialist approach to ‘development’ and a dismissal of critical race studies that caused considerable questioning within the field [8,9]. Ferretti [10] continued this critique, emphasizing recent calls for plurality and inclusiveness (from [11]), and on deconstructing past colonialist approaches [10,11]. Viles [12] also shared a critique of geography’s analytical capacities, remarking how geographers were seen as ‘lesser than’ other hard sciences, despite Holt-Jensen later asserting that geography was traditionally the only discipline to bridge the social and natural sciences (an important note to consider in our later discussion of environmental influences on health) [6].

Sharpe [13] introduced concerns that the discipline risked muddying its identity with ‘excessive pluralism’, remarking on the field becoming dominated by ‘hyphenated geography’. Encouragingly, Sharpe also quotes Peet in saying that “geography has a permanent identity crisis because what geographers do is complex” [13,14] (p. 1). Skole likewise offered reassurance that the field retained a consistent identity as a ‘melting pot’ of disciplines, able to incorporate theoretical and conceptual advances from those fields into its overarching mission [15]. Similarly, Holt-Jensen called geography a ‘science of synthesis’ [6].

Yet, as geographers entered the period in which GISci or geographic information systems (GIS) would make up an increasingly large part of the discipline, there became a “sense that other fields have been “encroaching” on geography by adopting many perspectives and approaches without giving credit” [5] (p. 498). Additionally, this in spite of geographers being “a breed inclined to wandering, exploring, and working together with others” (p. 497). Importantly, Baerwald also remarks that geography is not a corral or a toolbelt, but a ‘big tent’ discipline [5]. This is particularly important to keep in mind when considering that one must not use GIS to explore inequality, socioeconomic status, culture, or health behaviors from a geographical perspective. Despite the internal wrestlings that geographers have undertaken, there appears to be an evolution of the discipline along a consistent spine of interrogating space and place in order to better understand society, so that appropriate solutions can be explored. Additionally, although concerns have been raised over ‘hyphenated geography’, many of geography’s methodological and empirical advances have come from the sub-field central to this paper: medical geography.

## 3. The Growth of Medical Geography and GIS

While one can make delineations between the evolutions of medical geography and spatial or environmental epidemiology, they share certain elements in common which place them apart from other medical and health research. One aspect that binds them together is the growth of the field of GISci—and its associated theories and frameworks—around the tools of GIS.

Medical geography can rightly make claims to sharing a provenance with John Snow’s work on cholera [16,17]. Snow, of course, illustrated the location of cholera cases in London with a map as a communication tool, though he had previously established the root of the outbreak as coming from a specific pump. This was one of the first (if not the first) cases of using GIS to examine how the environment was connected to public health. This thinking was new at the time, and it is something medical geographers and physicians alike use in grounding their understanding of place and health.

More specifically to contemporary work, discussion of medical geography’s place in academia and society dates back at least to the post-war era and Jacque May’s seminal work on the ecology of disease [18]. May offered medical geography as a distinct sub-field requiring that special attention be given to time and space, alongside proposing the use of the now seldom-used concept of ‘geogens’, which are thought to contribute to disease. May continued in presenting the importance of a medical geographical approach by pointing out the various ways that culture and the environment influence disease onset and how variations in rates of disease by place necessitate a geographical understanding. This was highlighted again by Eyles and Woods, who stated the importance and need to examine both the social and cultural aspects of health determinants and the burden of disease [19].

Meade continued this line of questioning, discussing how the introduction of a cultural element into disease provided a basis for geographers to insert themselves into its study, and how this may separate their work from that of epidemiologists [16]. Meade went on to say how disease was now thought of as “the maladaptive interactions among the familiar triad of population, environment, and culture” [16] (p. 382). Meade also laid out important concepts related to daily exposures and activity spaces that ring relevant to work today. Yet, even by the 1980s, medical geography remained on the periphery of the medical sciences [20]. Even so, Mayer highlighted the critical independent importance of medical geography in shaping understanding of the spatial distribution of disease and health risk factors, and how continued interdisciplinary work would be necessary to help realize its potential.

The advent of separate medical geography symposia highlighted the growing role of the subdiscipline, which at the time was focused along two streams concerned with disease ecology and healthcare provision [21]. The field is described as one with applied, rather than theoretical, roots. In the first issue of the now-key journal *Health & Place*, Powell points out many of the same problems that beset place-based medical research: that the influence of place is either poorly conceptualized, or ignored altogether [22]. Yet, Powell also offers an important lesson: that geography will sometimes be a relatively unimportant factor, and we should not be so concerned with spatial variation that we overlook aspatial factors. Kearns also stated the need for reforming or modifying medical geography with a focus on the socio-ecological model of health, which is now instrumental in environmental health research [23]. 

Linking Baerwald’s observation that GIS-based methods are increasingly being used by other disciplines alongside Hayes’ observation that we ought to use such tools to identify and address socio-spatial inequalities, geographers sit in a space where they simultaneously aim to educate and inform others about the value of a spatial approach and the associated tools of GIS, while highlighting the need for effective collaborations with geographers to leverage these tools appropriately [5,24]. Indeed, these ruminations and advances (of improving GIS-based methodologies and maintaining a focus on equity) make up some of the key underpinnings of GIS in health research.

GIS is essential to medical geography because of the power it affords to that basic concept of place and health first popularly illustrated by John Snow. Beyond observing patterns, GIS offers huge computational power to uncover invisible patterns, including moving beyond simple correlations to consider multi-level, longitudinal environmental exposures measured from meticulously measured activity spaces that account for the spatial polygamy of daily life [25]. GIS has many uses within the world of medical geography; it can be used to collect spatial data through aerial photography or remote sensing, analyze previously collected spatial datasets, or examine spatial patterns or hotspots within communities or across space. 

Many people do not understand how seminal or applicable GIS is to research on the environment and one’s health. Socio-spatial inequalities commonly relate to health outcomes and exposures in today’s world, where the application of GIS is the best way to examine inequalities and spatial patterns. The prevalence of inequalities in today’s world is well documented. For instance, cancer incidence and mortality are commonly higher among Indigenous Peoples in Canada, the United States, New Zealand and Australia [26,27,28,29]. A recent review article suggests it may not be just health outcomes that are more common among Indigenous Peoples, as Indigenous communities commonly have inequalities in exposure to pollutants in the environment as well [30]. This is commonly also the case in neighborhoods populated by Black and other minoritized population groups. The coincidence of health outcomes in space links closely to Soja’s theory of thirdspace, which posits that not only is space represented by coordinates, but in the functional and perceptual aspects that define who lives there, what functions a space serves, and how individuals interact with an reproduce space [31].

Yet, the practice of GIS in medical geography should not be undertaken without keen understanding of the methodological and theoretical issues involved in its use, and common to geography more broadly. Sadler and Lafreniere lay out a series of considerations that non-geographers should keep in mind when working with geographic data, ultimately leaning on the suggestion that collaboration is key to leveraging the power of GIS [32]. The expertise brought to bear by geographers often helps avoid common pitfalls such as the modifiable areal unit problem, ecological fallacy, the edge effect, and others. However, geographers also bring analytic expertise in spatial statistics not well-mimicked by other disciplines. In effect, spatial science research initiated by those not trained in GIS often lacks a depth of understanding that prevents effective discovery of spatial patterns, or worse, false identification of patterns where none exist. Thus, the presence of GIS and involvement by trained spatial scientists has multiplied the possibilities for geography in medical and public health research. Additionally, qualitative insights gathered from focus groups and other grounded research can meaningfully complement the more quantitative aspects of medical geography. The perspectives geographers bring have also played a role in advancing the terminology and methodology available to these fields.

## 4. Precision of Language in Exposure Classification

We earlier presented the centrality of place in geography, and that the approaches taken to studying place distinguish geography as a discipline. Building from this, we offer that the very approaches taken by geography afford a necessary precision to the work of public health research and the elucidation of public health concepts. Without the ability to accurately interrogate and define concepts related to access and exposure to geographical phenomena, the study of whole areas of research would be methodologically imprecise.

Take, for example, the concept of ‘food deserts’, itself a well-critiqued term within planning and public health research. Without a geographical framework, the concept of where communities have relatively better or worse food access would be (and often is) diminished to easily observable features (e.g., is there a grocery store nearby?). A line demarcating relatively better from worse food access could not be drawn. However, indeed, that has been the case in many investigations of food access, as discussed in several recent reviews [33,34,35,36]. The first version of a food desert atlas by the USDA was plagued by the same fundamental issues of poor data quality, simplistic geographic measures, and inappropriate geographic scales, and some issues remain in the current version [37]. Yet, the involvement of geographers in food access research has helped move the field toward considering the influence of daily activity spaces [38,39] and the influence of quality, variety, and price of goods in all types of stores [40,41].

Research on tobacco and alcohol outlet density (AOD) is going through a similar evolution: earlier studies examined the total number of outlets per geographic unit or population, or the used of straight-line buffers around sites [42,43]. Additionally, guides created by major organizations such as the CDC offer little help in the way of truly interrogating the complexities of alcohol outlet exposure [44]. Recent work and reviews by geographically grounded researchers have helped AOD research move meaningfully toward where food access research finds itself, by considering the availability of products in stores, the hours of operation, and more sophisticated spatial access-based analysis methods [45,46,47,48,49].

One might also consider the conceptualization of area-level poverty. Many public health studies have examined ‘neighborhood disadvantage’—which could variously be a stand-in for vacant homes, disorder, crime, environmental exposure, or many other deleterious characteristics. Yet, in many studies on this topic, disadvantage is merely a synonym for census tract- or ZIP code-level poverty rates, with no consideration given to local-level data that could more accurately represent multiple dimensions of disadvantage [50,51,52,53]. The finer approach offered by a geographical lens enables compilation and distillation of a greater number of metrics, as well as a more nuanced scale of measurement (e.g., kernel density estimates, values obtained via areal interpolation), as in Sadler et al. [54].

Examining environmental pollution and exposure has a similar complexity that is missed without geographical expertise. Indeed, at the outset of media attention on the Flint (Michigan) Water Crisis in 2015, state level officials sought to discredit the researchers raising the alarm because their team lacked sufficient knowledge of the modifiable areal unit problem [32,55]. Such critical expertise is likewise necessary in measuring the potential impacts from climate-change-induced natural disasters, water bodies, or landforms [56].

In each of these examples, complex social or environmental phenomena (food access, alcohol outlet density, neighborhood disadvantage) are often reduced to overly simplistic representations, which limits the confidence of inference that can be made. The contribution of geographers is to think more deeply and model with more sophistication additional ways to conceive of these concepts.

## 5. Known Linkages of Exposure to Health

With these examples in mind, it bears illustrating additional ways that geographers define exposures and access, much of which is performed in the study of exposure science. Medical geographers are centrally concerned with how the environment may relate to various health behaviors and outcomes [49,57,58,59,60,61,62]. Environmental exposures can come from natural features such as the air, water, or sun, but can also relate to neighborhood features around one’s home, workplace, or school. 

For instance, access to parks around the home can influence physical activity levels or mental health [63,64]. A home environment with fewer fast-food restaurants and more recreational opportunities may help to reduce obesity levels in children [58]. Similarly, a higher density of healthy food outlets or supermarkets around the home may relate to a lower body mass index (BMI) among children [65], and can moderate the association between food insecurity and poor mental health [66]. Relatedly, social connectedness has been shown to moderate the impact of vacancy on mental health [67].

Tobacco and alcohol retailers are well known to target urban communities [68], and increased density of such retailers leads to higher rates of use [69]. Similarly, for adolescents, fewer tobacco outlets around high schools may lead to lower smoking rates [49]. However, poorer-quality built environments overburdened by alcohol outlets driven by structural racism can lead to higher rates of heavy drinking [70,71].

Certain characteristics of the built environment such as sidewalks, street connectivity, residential density, retail space, and land use mix may also relate to walkability or overall physical activity [72,73,74,75,76], while the environment may influence children in different ways. Adults may be inclined to walk to shops, cafes, restaurants, or other retail destinations, meaning land use mix and street connectivity are important. Children or their parents, conversely, may have concerns over safety, so aspects such as fewer vehicles, the number of street crossings, and busy streets while traveling to school may encourage more active modes of transport such as walking and cycling [77,78,79]. 

Beyond behavior, environmental exposures may also influence health. Air pollution is composed of a complex mixture of agents including numerous carcinogenic and dangerous substances that may influence systemic and chronic inflammation, stress, and DNA damage to tissues throughout the human body [80]. Particulate matter (PM), a component of air pollution, is a known human lung carcinogen [80,81,82,83] and is connected to adverse health effects [84]. Ambient air pollution may relate to higher cancer risks for certain populations [85] or certain respiratory conditions such as chronic obstructive pulmonary disease (COPD) [60]. Potential exposures to air pollution vary widely by spatial location. Examining how air pollution may relate to health outcomes across space is another example of how medical geography can add to the scientific discourse. 

Other prevalent exposures in everyday life are radiation-based and known carcinogens, including ultraviolet (UV) radiation and radon. Varying levels of solar UV exposure are present in the outdoor environment, and has been linked to skin cancer [86]. Overall exposure can vary by geographical location, season, weather and time of day, but also by the time spent outside and the amount of skin exposed [87]. Another example, radon, is a naturally occurring radioactive gas released through rocks and soils as uranium breaks down [88]. Radon is the leading cause of lung cancer in non-smokers [88,89]. Radon exposures vary based on individual characteristics such as age, sex, and genetics, along with time spent at home [90,91,92,93], but also relate to the built environment. Changes in building practices have led to higher levels of radon accumulating in homes and other buildings [94,95]. The exposure within the residential environment is concerning, since most people in North America spend nearly 70% of their time in their homes [96].

Pesticides are another common exposure that relates to where you live. Pesticides are very common in modern agricultural production as they can help to increase productivity, maximize crop productivity and also just make the overall crops more aesthetically appealing [97,98]. The issue here is that pesticides applied to crops also enter the environment (soil, water, and air) and can travel beyond the original application area [99,100]. Exposure to pesticides within the general population happens through diet contact with contaminated air, water and soil [101]. Studies have recently adopted GIS-based methods to estimate exposure and have reported that people residing in close proximity to agricultural land commonly have higher levels of pesticides in their environment [85,102].

Environmental exposures influence behavior in different ways, and not everyone will react the same to such exposures. Importantly, however, medical geography allows for the examination of these spatial differences within the environment. It provides the tools and methods necessary to examine spatial variations in potential exposures and health behaviors.

## 6. ‘Hotspots’ and ‘Clusters’

One methodological request medical geographers commonly receive—especially from community partners—is to look for hotspots, or clusters. In reality, these are more challenging to actually find than one might suspect, especially for cancer and other less common or more complex health outcomes, and for diseases that have strong genetic predispositions (as discussed in [103,104]). People unacquainted with the science behind this work may become concerned when neighbors are affected by cancer. If they live near a known pollution source or contaminated site, they may initiate a cancer cluster investigation with a simplistic assumption that a singular exposure caused a singular outcome. Yet, it is generally very rare that cluster investigation methods are able to detect a true cluster of disease [105]. One example comes from the United States, where one review found that only 1 of 428 suspected clusters was statistically significant [105]. Reasons for this gap include the inability to control lifestyle factors, unknown exposure timelines, multiple exposure settings, occupational exposures, various exposure–cancer site pairings, unknown spatial scales, and small populations [106]. Paramount to all of these is the well-studied issue of spatial dependence, which—in concert with Tobler’s First Law of Geography—holds that phenomena closer in space are more likely share characteristics (as explored in [107]). Regardless, the exploration of clusters or hotspots has been and will continue to be a challenge in environmental health given the lack of data on exposure and latency.

One of the biggest challenges when examining exposure is measuring how and where people are potentially exposed. Spatial methods used by medical geographers are key in this instance. Understanding how people are differentially exposed is instrumental to this exposure science research. Methods for accessing exposure are typically through self-report or using a combination of GIS and/or global positioning systems (GPS) to estimate exposure [85,108,109]. The technological improvements and use of GIS/GPS-based assessments allow for more realistic locations of what environments people are exposed to than self-report [110,111]. These methodological approaches are nested into the history of medical geography and can be identified in Dr. Snow’s dot map of cholera cases in London. While technology has and continues to change, the importance of spatial methodology holds true and is always changing to improve how we measure potential environmental exposures across space.

While it is easy to highlight the importance of GIS and spatial-based methods, it is also necessary to understand some issues and critical decisions that need to be made. Foremost, understanding the biological and behavioral antecedents to disease is essential to adequately accounting for other potential causes. Thus, it would be inadequate to merely approach an issue with GIS-the-tool in-hand without a theoretical and conceptual understanding of the interplay of behavior, genetics, and environment. Furthermore, occupational exposures are another important avenue for potential exposure with links to illness and disease. These occupational environments must be accounted for outside of the GIS-based or environment-based analyses.

Additionally, we previously mentioned issues associated with ecological fallacy and the modifiable areal unit problem; these are significant limitations in all ecological-based spatial research projects. Using a different sized spatial unit will commonly relate to varying results, and it is important for medical geographers (or others performing the research) to ensure they are using a representative spatial unit. Testing of various sized units, sensitivity analysis, or other approaches are key to ensuring results are representative. Similar thoughts and approaches also need to be considered when areal-based units are trying to represent individual behaviors, confounders, or outcomes. Finally, more subjective measures of GIS analysis such as buffer distance or buffer types also need to be considered. Common approaches such as using both density and proximity measures help to more accurately capture access and exposure within one’s environment.

## 7. Linking to the Medical Field

The authors have spent the entirety of their post-doctoral careers in the world of medicine, healthcare, and public/population health. As such, the work we have taken on has distinguished us from our colleagues residing in geography departments and our colleagues in the medical/public health fields. Many of the inquiries noted above take place in isolation from the medical field directly, yet in many more domains we have observed incidental and purpose links to the medical field. Examples of linkages may include geocoding of participants homes, incidences (i.e., collisions), or other spatial locations where the environment can be analyzed. In most cases, we are called upon to be methodological experts, lending expertise to topics including CPR education [112], trauma transfer protocols [113], access to health care [114], bystander CPR [115], spatial analysis of air pollution and asthma [60], autism support services provision [116], physical activity guidelines for cancer survivors [117,118], buprenorphine treatment facility siting [119], lead exposure [120] and pedestrian safety [121,122].

The implications of having these connections reside in the added opportunity to work with protected health information from patient records (the most obvious connection being to geocode them). Whereas those without connections to the medical field must rely more closely on primary data collection methods to conduct human subjects research, the ability to pull from medical records opens many doors. Retrospective, longitudinal cohorts have been pulled in studies of historic built environments, while contemporary intervention studies have the possibility to be leveraged with contemporary data to uncover similar environmental influences. This is not to discourage the ongoing work of longitudinal work outside of medical domains, but rather to raise awareness to the possibilities of collaborative approaches that can leverage such datasets.

These collaborative efforts are critical to both medical geographers but also physicians and public health researchers. While medical geographers benefit from the access to data, it is mutually beneficial. As previously stated, there are many important decisions that need to be made and addressed related to using and analyzing spatial data. If the wrong approaches are taken, the study and results will be limited, and the overall study will be methodologically flawed.

## 8. Conclusions: Making a Case for Geography: Replicable/Adaptable Approaches

Given our work within and beyond the medical field, one of our central concerns continues to be making geographical work useful and relevant to the medical community, while at the same time conducting methodologically rigorous work in our field.

Beyond merely ‘making maps’, the geographer plays a critical role in thinking about, conceptualizing, and interrogating space and place (and how to represent them). However, many initial requests for collaboration can reduce our work to being glorified (carto)graphic designers and geocoders, whose analytic skills are reduced to geocoding points in space and making attractive maps to illustrate simplistic concepts of location and coincidence of phenomena in space. While most enjoy the ‘pretty map’ that was made, there is a lot of thought that needs to go into the map design. One of the first questions that we ask ourselves is should we even make this map? What will it show? Will it accurately represent reality, or will it cause more harm and confusion? These are important questions as our job and discipline should not be to merely make a map. The difference between this and a good collaboration is when we actually provide input on the spatial analysis, methodology, outcome and even data collection as needed. 

These steps are crucial to performing meaningful collaborations with colleagues across the interdisciplinary world that medical geography is nested in. Just as community partners being present in research from the beginning offers added dimensions for question generation, data collection approaches, and analysis techniques, so too does the engagement of geographers in medical research. Most straightforwardly, ensuring that geographic data are collected from medical records or studies increase the value of a geographer’s contributions. Asking perception or experiential questions about one’s home, school, or workplace environment likewise affords a depth to such work. Additionally, having a geographer at the table means looking for relationships and causes outside of the individual.

The involvement of other disciplines in medical research is becoming more common. Given geography’s role as a highly interdisciplinary subject combining natural and social science, it has been well-equipped with appropriate tools and theories to study this nexus. On the flip side, community-focused medical schools train up future physicians to understand the social determinants of health and other concepts that take the study of human health outside the body. As more physicians come to recognize the interplay between humans and their environment, and as GIS continues to become more commonly understood as a research tool, the rationale for including geographers in such work will be strengthened. Ultimately, regardless of the status of geography or medical geography as a discipline or subdiscipline, the tools used by geographers necessitate real expertise and offer real opportunities for collaboration. Identifying these potential missed opportunities will hopefully make future research endeavors more attuned to the possibilities of such a collaboration.
